# Supplementary material for: Krill Oil Inhibits NLRP3 Inflammasome Activation in the Prevention of the Pathological Injuries of Diabetic Cardiomyopathy
Source: Nutrients. 2022 Jan 15;14(2):368. doi: 10.3390/nu14020368 (PMC8780413; doi:10.3390/nu14020368)
Supplement: Supplementary file 1 [file nutrients-14-00368-s001.zip › nutrients-1521602-supplementary.pdf]

Supplementary Material

Table S1. Ingredients composition of experimental diets.

Ingredient (g/kg)	Normal diet	High-fat diet	Krill oil
Soybean oil	70	70	55
krill oil	-	-	15
Lard	-	230	230
β-Cornstarch	397.49	110.62	110.62
α-(dextrinized) Cornstarch	132	36.87	36.87
Casein	200	250	250
Sucrose	100	200	200
AIN-93 mineral mixture	35	35	35
AIN-93 vitamin mixture	10	10	10
L-Cystine	3	3.75	3.75
Choline bitatrate	2.5	2.5	2.5
Cellulose	50	50	50
tert-Butylhydroquinone	0.01	0.06	0.06
Cholesterol	-	1.2	1.2
Protein	203	253.75	253.75
Carbohydrates	629.49	347.49	347.49
Fat	70	300	300

Table S2. Composition analysis of krill oil used in the study

Krill oil	Result	Unit
Astaxanthin	232.15	mg/kg
Peroxide	< 0.2	eEq/kg
Saponification	163.0	mg/KOH/g
Total phospholipids	55.06	g/100g
Fatty acid composition		%/total fatty acids
C14:0	10.51	
C16:0	25.77	
C16:1	6.15	
C18:0	0.28	
C18:1	14.2	
C18:2	1.33	
C18:3	1.59	
C20:3	1.19	
C20:4	0.73	
C20:5 (EPA)	22.89	
C22:6 (DHA)	13.83	

Table S3. Sequences of primers used for qRT-PCR.

gene name	Sequences of primers	GenBank accession number	Product size
Actα2-F	TAACCCTTCAGCGTTCAGC	NM_007392.3	99
Actα2-R	ACATAGCTGGAGCAGCGTCT	NM_007392.3	99
Ccl2-F	CTTCTGGGCCTGCTGTTCA	NM_011333.3	127
Ccl2-R	CCAGCCTACTCATTGGGATCA	NM_011333.3	127
Col1α1-F	CCGCTGGTCAAGATGGTC	NM_007742.4	117
Col1α1-R	CTCCAGCCTTTCCAGGTTCT	NM_007742.4	117
Gsdmd -F	TGTCAACCTGTCAATCAAGGA	NM_026960.4	68
Gsdmd -R	AGCCAAAACACTCCGGTTC	NM_026960.4	68
Icam1-F	GCTACCATCACCGTGTATTCG	NM_010493.3	80
Icam1-R	AGGTCCTTGCCTACTTGCTG	NM_010493.3	80
Ifn-γ-F	AGCGGCTGACTGAACTCAGATTGTAG	NM_008337.4	247
Ifn-γ-R	GTCACAGTTTTCAGCTGTATAGGG	NM_008337.4	247
Il-1β-F	GACCTTCCAGGATGAGGACA	NM_008361.4	183
Il-1β-R	AGCTCATATGGGTCCGACAG	NM_008361.4	183
Nox4-F	TTGCCTGGAAGAACCCAAGT	NM_001285835.1	84
Nox4-R	TCCGCACAATAAAGGCACAA	NM_001285835.1	84
Rplp0-F	AGCAAAGGAAGAGTCGGAGG	NM_007475.5	83
Rplp0-R	GGCTGACTTGGTTGCTTTGG	NM_007475.5	83
Sele -F	TCAGTGGCTGCCGAAGTATT	NM_011345.2	69
Sele -R	TGGATCTCATGCTGGCTTCA	NM_011345.2	69

Sirt3-F	TCGTCCTGCAGAGCATCAT	NM_001127351.1	106
Sirt3-R	ACAGAGGGATATGGGCCTTC	NM_001127351.1	106
Tnf-α-F	TTGTCTTAATAACGCTGATTTGGT	NM_001278601.1	61
Tnf-α-R	GGGAGCAGAGGTTCAGTGAT	NM_001278601.1	61

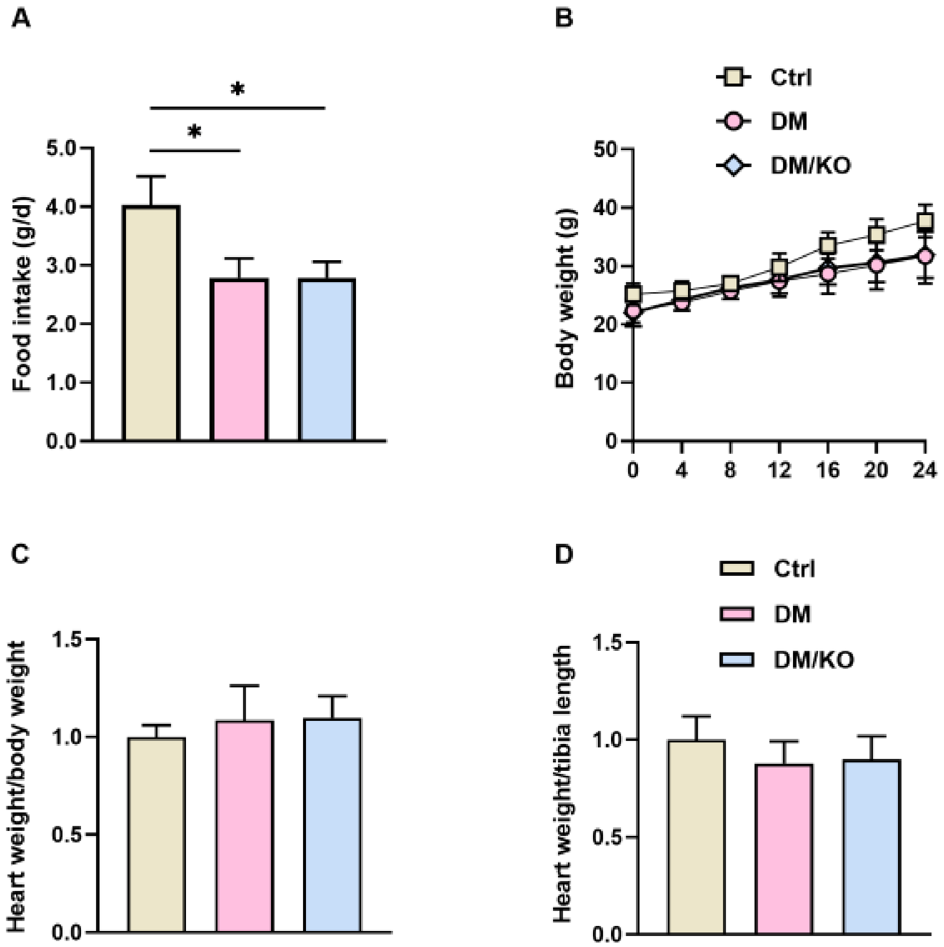

Figure S1. Basic data for animal models (**A**) Average food intake. (**B**) Body weight. After harvesting, the hearts were weighed, with ratios of (**C**) heart to body weight, and (**D**) heart weight to tibia length calculated. For (**C**) and (**D**), the data were normalized to Ctrl, and summarized as means ± SD. *, $P < 0.05$ vs. Ctrl; Abbreviations: Ctrl, control; DM, diabetes mellitus; KO, krill oil.
